# An evaluation of the chemical content and microbiological contamination of Anatolian bee venom

**DOI:** 10.1371/journal.pone.0255161

**Published:** 2021-07-22

**Authors:** Aslı Elif Tanuğur-Samanc, Meral Kekeçoğlu

**Affiliations:** Department of Biology, Düzce University, Düzce, Turkey; King Khalid University, SAUDI ARABIA

## Abstract

Bee venom is a natural substance produced by worker bees. The aim of this research paper is to determine the characteristics of Anatolian bee venom by evaluating its chemical content and microbiological properties. Physical, chemical and microbiological analyses were performed on 25 bee venom samples from different areas of Anatolia, Turkey. Data obtained by 3-replicate studies were evaluated with normality and one-way and two-way ANOVA / Tukey tests. Chemical analyses of the bee venoms revealed average melittin, apamin, and phospholipase A2 contents of 40.57%, 2.12% and 13.67%, respectively. The results suggest that Anatolian bee venom has a high phospholipase A2 content compared to the previous literature. The results for apamin content were similar to those reported in other countries. Melittin content was within the range of standard values. Bee venom samples were also observed to have a high sugar content, associated with pollen and nectar contamination. Total aerobic mesophilic bacteria counts revealed no microbial development in 11 samples of bee venom. *Staphylococcus aureus* was not detected in any sample. A low microbial load was associated with a high phospholipase A2 content in the bee venom composition, thus contributing to its antimicrobial character. This study presents an examination of Anatolian bee venom in terms of chemical content and microbial quality. The examination of other components in addition to phospholipase A2, melittin and apamin in future studies, together with an analysis of antimicrobial properties will further our understanding of Anatolian bee venom.

## Introduction

Bee venom is a natural substance produced by worker bees (*Apis mellifera anatoliaca*) [1]. Honeybees have two separate glands at the base of the needle apparatus, an acid gland (venom gland) and an alkaline gland (Dufour gland). The venom gland is responsible for producing bee venom and thus plays a defensive role. The venom produced is stored in the venom gland [2]. This gland begins to produce bee venom with the emergence of the new adult bee [3]. Although one-day-old bees possess some quantity of venom, they are unable to sting during this period since the sting has not yet hardened. From the second day of life on, the activity of the acid gland increases, and bee venom production peaks in bees aged 16–19 days. The amount of venom in a bee ranges between 0.05 and 0.3 ml / bee, depending on the season and the nature of the animal [2].

Bee venom consists of a complex mixture of proteins, peptides and low molecular components [1]. It is also known for its antibacterial, anti-fungal, antiviral, metabolic, anti-inflammatory, anti-arthritis and anticancer characteristics. Bee venom exhibits numerous biological activities, such as effects on the immune system, the central and peripheral nervous systems, and the cardiovascular system [4]. In addition to being traditionally used in the treatment of pain, skin diseases and rheumatism, studies have also reported that it exhibits anti-carcinogenic activity against prostate, liver and breast cancers [5].

Melittin, which constitutes 40%-50% of the dry weight of bee venom, is a polypeptide with a 26 linear amino acid sequence. Melittin, which is cationic and hemolytic, exhibits amphipathic properties by containing hydrophilic parts with the carboxy end and hydrophobic parts with the amino side in its structure [6]. In addition to reducing surface tension by disrupting the membrane structure, it also induces cortisol production with its anti-inflammatory effects. It affects the central nervous system and enables the pituitary gland to produce cortisol. Melittin increases blood circulation by affecting the muscles and causes a decrease in blood pressure [7]. One study of different bee venoms examined the effects of bee venom on gram-positive and gram-negative bacteria and reported that melittin contributed to the anti-bacterial properties of the venom [8].

Bee venom is used in the treatment of immune-related diseases and tumours [9, 10]. Peptides in the venom, such as melittin and phospholipase A2, target numerous cancer cells such as renal, lung, liver, prostate, bladder and breast cancer cells [11]. Moreover, it has been suggested that the anti-cancer mechanism of action of bee venom is due to the cytotoxic effect in the cell caused by the activation by melittin of phospholipase A2 [12, 13]. Melittin exhibits effects on tumour cells. Studies show that melittin inhibits growth in tumour cells by stopping the cell cycle. In addition to changing the membrane structure of cancer cells and preventing their transformation, it also causes cells to undergo apoptosis, programmed cell death. In one study, melittin was tested for apoptosis of tumour cells in liver cancer (HCC), one of the most common types of cancer worldwide. Positive results were obtained with its use in combination with various compounds [14]. Melittin is thought to exert a similar effect to that on tumour cells on other types of cancer cells. In another study involving HIV-1, melittin was found to prevent transmission of the virus. While toxic effects were observed on cells involving free-form melittin, this effect disappeared when melittin was applied as a nanoparticle, and it exhibited anti-viral properties [15]. It is therefore important for bee venom samples to have a high melittin content, since this determines the venom quality.

In contrast to other substances, phospholipase A2 in bee venom is an enzyme and is present at levels of approximately 10% - 12% by dry weight. This enzyme, the most effective of the known allergen substances in venom, is also a harmful component that causes the breakdown of phospholipids. It also breaks down the phospholipids in the cell membrane, causing the membrane structure to deteriorate. Researchers have concluded that the enzyme homologous to human phospholipase A2 inhibits cell death caused by prions [16]. While the enzyme activated by melittin is thought to be involved in nervous system diseases such as Alzheimer’s and Parkinson’s, it also affects the development of inflammation. In response to these effects of phospholipase A, it has been determined that phospholipase B, which enables the breakdown of toxic components, exhibits activity in bee venom [17].

Apamin, which contains 18 amino acids, contains two disulphide bridges in its structure. One of the most important properties of apamin, one of the smallest neurotoxins in the venom, is its anti-inflammatory effect. An experiment on mice with atherosclerosis, a chronic inflammatory disease, showed that apamin can be used in the treatment of the condition, with dose-dependent anti-inflammatory effects [18]. In addition, while low doses affect the central nervous system, high doses cause neurotoxic effects [19]. Apamin exhibits an allosteric inhibitor property playing a significant role in several pathophysiological responses, such as atherosclerosis, Parkinson’s disease and hepatic fibrosis [20–23]. Apamin has been shown to exert a selective blocking effect on Ca^+2^ dependent K^+^ (SK channels) channels in the central nervous system. It has also been proposed for use in the treatment of Parkinson’s disease due to this effect, which is beneficial for repetitive movements in neurons [19]. High concentrations of apamin have been associated with an increase in pro-inflammatory cytokines [24]. The chemical content of bee venom is therefore highly important in terms of its efficacy. The purpose of this paper was to investigate the content and microbiological contamination of bee venom samples obtained from different parts of Anatolia, Turkey. Within the scope of the study, the venom and infrastructure were first prepared for the analysis, followed by the development and validation of the analysis methods. Since bee venoms may differ from each other depending on the region and time of collection, 25 different bee venom samples were obtained from different parts of Anatolia. These were then subjected to physical, chemical and microbiological analyses.

## Materials and method

### Sample collection

Twenty-five bee venom samples were collected from different cities in Turkey by an Apimak bee venom collector (Arsum, Turkey) from the entrance of the hives. The bees passing through this layer insert the glass where they are exposed to an electric current. Once the venom has dried in this glass layer, it is collected by the beekeepers. The times and places of bee venom sample collection are given in Table 1.

**Table 1:** Times and places of collection of bee venom samples.

Sample	Origin	Year of collection
Bee Venom 1	Manisa	June 2019
	(Located between 27˚ 08’ and 29˚ 05’ east longitude and 38˚ 04’ and 39˚ 58’ north latitude.)	
Bee Venom 2	Malatya	June 2019
	(Located between 38° 45’ and 39° 08’ east longitude and 37° 54’ and 39° 03’ north latitude.)	
Bee Venom 3	Adana	June 2019
	(Located between 35° 52’ and 36° 42’ east longitude and 36° 57’ and 37° 45’ north latitude.)	
Bee Venom 4	Adana	July 2019
	(Located between 35° 52’ and 36° 42’ east longitude and 36° 57’ and 37° 45’ north latitude.)	
Bee Venom 5	Balıkesir	July 2019
	(Located between 26° 28’ east longitude and 39° 40 ’north latitude.)	
Bee Venom 6	Denizli	August 2019
	(Located between 28° 30’ and 29° 30’ east longitude and 37° 12’ and 38° 12’ north latitude.)	
Bee Venom 7	Muğla	August 2019
	(Located between 27° 13’ and 29° 46’ east longitude and 36° 17’ and 37° 33’ north latitude.)	
Bee Venom 8	Malatya	July 2019
	(Located between 38° 45’ and 39° 08’ east longitude and 37° 54’ and 39° 03’ north latitude.)	
Bee Venom 9	Adana	July 2019
	(Located between 35° 52’ and 36° 42’ east longitude and 36° 57’ and 37° 45’ north latitude.)	
Bee Venom 10	Adana	July 2019
	(Located between 35° 52’ and 36° 42’ east longitude and 36° 57’ and 37° 45’ north latitude.)	
Bee Venom 11	Denizli	July 2019
	(Located between 28° 30’ and 29° 30’ east longitude and 37° 12’ and 38° 12’ north latitude.)	
Bee Venom 12	Denizli	June 2019
	(Located between 28° 30’ and 29° 30’ east longitude and 37° 12’ and 38° 12’ north latitude.)	
Bee Venom 13	Manisa	July 2019
	(Located between 27˚ 08’ and 29˚ 05’ east longitude and 38 04’ and 39˚ 58’ north latitude.)	
Bee Venom 14	Muğla	July 2019
	(Located between 27° 13’ and 29° 46’ east longitude and 36° 17’ and 37° 33’ north latitude.)	
Bee Venom 15	Konya	June 2019
	(Located between 31° 14’ and 34° 26’ east longitude and 36° 41’ and 39° 16’ north latitude.)	
Bee Venom 16	Adana	August 2019
	(Located between 35° 52’ and 36° 42’ east longitude and 36° 57’ and 37° 45’ north latitude.)	
Bee Venom 17	Malatya	June 2019
	(Located between 38° 45’ and 39° 08’ east longitude and 37° 54’ and 39° 03’ north latitude.)	
Bee Venom 18	Malatya	August 2019
	(Located between 38° 45’ and 39° 08’ east longitude and 37° 54’ and 39° 03’ north latitude.)	
Bee Venom 19	Denizli	July 2019
	(Located between 28° 30’ and 29° 30’ east longitude and 37° 12’ and 38° 12’ north latitudes.)	
Bee Venom 20	Adana	July 2019
	(Located between 35° 52’ and 36° 42’ east longitude and 36° 57’ and 37° 45’ north latitude.)	
Bee Venom 21	Denizli	July 2019
	(Located between 28° 30’ and 29° 30’ east longitudes and 37° 12’ and 38° 12’ north latitude.)	
Bee Venom 22	Adana	June 2019
	(Located between 35° 52’ and 36° 42’ east longitudes and 36° 57’ and 37° 45’ north latitude.)	
Bee Venom 23	Muğla	July 2019
	(Located between 27° 13’ and 29° 46’ east longitude and 36° 17’ and 37° 33’ north latitude.)	
Bee Venom 24	Konya	June 2019
	(Located between 31° 14’ and 34° 26’ east longitude and 36° 41’ and 39° 16’ north latitude.)	
Bee Venom 25	Manisa	June 2019
	(Located between 27˚ 08’ and 29˚ 05’ east longitude and 38˚ 04’ and 39˚ 58’ north latitude.)	

### Physical analysis in bee venom

Bee venoms were analysed by examining their smell and appearance. In order to remove impurities, 0.1 grams of powder bee venom samples were dissolved in 10 grams of distilled water and filtered. Foreign material and other impurities were removed by filtration (Whatman® qualitative filter paper, Grade 1).

### Chemical analysis in bee venom

#### Determination of bee venom content by high performance liquid chromatography-ultraviolet light (HPLC-UV)

Melittin, apamin and phospholipase A2 in the bee venom samples were separated using HPLC (VWR International, Radnor, USA) in a suitable solution diluted with water, and subsequently determined photometrically in the ultraviolet region. A Supelco Supelcosil LC-318 (Sigma-Aldrich Co. LLC, Darmstastadt, Germany) column (4,6 x 250 mm, 5 *μ*m) was used at 220 nm. Ultra-distilled water and 0.1% trifluoroacetic acid (TFA) were used for mobile phase A and 80:20 acetonitrile (ACN), ultra-distilled water and 0.1% trifluoroacetic acid for mobile phase B. The linear gradient 24 method was applied with 5%-80% mobile phase B. The flow rate was 1 ml / minute and the injection volume 40 μl [25].

Analyses were performed using the modified DIN 10758 method. Stock standard solutions of 1 mg / ml were prepared by dissolving phospholipase A2 and melittin in ultra-pure water and apamin in ultra-pure water with 0.05 M acetic acid. By taking the appropriate amount from each solution, standard solutions of 2%, 5%, 10%, 20%, 40%, and 50% were formed and fed into the vials and given to the HPLC device, where calibration curves were created. Next, 5 mg of bee venom samples to be analysed were weighed and diluted with 10 ml of ultra-pure water. The syringe tip was filtered, placed into the vial, and given to the HPLC device. Three replicates were analysed from each sample. Quantification was performed according to the external standard method, using peak areas and peak heights [26].

Sugar analyses in the bee venom samples were performed with a Thermo Fisher Scientific APS-2 Hypersil (Thermo Fisher Scientific Inc., Waltham, USA) column (4 x 250 mm, 5 um) in HPLC using a Refractive Index (RI) detector. Isocritical analysis was carried out using the acetonitrile:ultra-pure water (80:20) mobile phase. Standards for fructose, glucose and sucrose were obtained from Sigma Co. Sugars in 0.5 g bee venom sample were extracted with acetonitrile-water solution and Carrez I-II and then purified by centrifugation and filtration [26].

#### Moisture content

Moisture determination analysis was carried out at 105°C with a Precisa XM50 (Precisa Gravimetrics AG, Switzerland) moisture analyser. Three analyses were repeated for each of the 25 different samples collected [26].

### Microbiological analysis

For total aerobic mesophilic microorganism counts, 0.1 grams of powder bee venom samples were weighed in a sterile container before being homogenized by adding 10 grams of distilled water into the sterile container. For optimal growth and recovery of microorganisms, the pH of the sample suspension was adjusted to 6.6–7.2, with 1N NaOH.1 mL sample being placed onto 3M™ Petrifilm™ aerobic count plates (3MCompany, USA) and left to solidify for 1 minute. The samples were than incubated at 35°C for 48 hours, and all red colonies were finally counted independently of size and colour density.

For *Staphylococcus aureus* ATCC ® 6538 (+) analysis, the samples were diluted by adding peptone salt and buffered peptone according to the ISO 6887–1: 1999 method, and then homogenized. The pH was adjusted to a range of 6–8 with 1N NaOH, and the samples were placed onto Petrifilm plates. Red-purple colonies were counted by incubation at 37° C for 24 hours.

In order to count *Candida albicans*, which can grow in simple culture medium or selective culture medium (CHROMagar Candida), the bee venoms were diluted and homogenized and placed onto Petrifilm plates. Counting was performed after incubation for 24–72 hours at 25–37° C.

For *Escherichia coli* ATCC ® 25922 Gram (-) counting, bee venom samples were first prepared and weighed into a sterile container. These were than homogenized by adding distilled water into the sterile container. For optimal growth and recovery of microorganisms, the pH of the sample suspension was adjusted with 1N NaOH to 6.6–7.2. Next, 1 mL of sample was placed on Petrifilm plates and left to solidify for 1 minute. Following incubation at 35° C for 24 hours, gaseous blue colonies were finally counted.

For total yeast and mould counts, the sample was weighed and placed in a sterile container. To this was added 0.1% peptone water and peptone salt diluents according to ISO method 6887–1. After homogenization, 1 mL was placed onto a Petrifilm plate and incubated at 20° C for 3–5 days, after which coloured regions were counted. Yeasts appear as small, bronze to blue-green coloured colonies, while moulds are large and brown, beige, orange, or blue-green in colour.

### Statistical analysis

The study data were subjected to statistical analysis. Data from repeated studies were evaluated with normality, one-way and two-way analysis of variance (ANOVA) / Tukey tests.

Melittin, apamin, phospholipase A2, glucose, fructose, sucrose and moisture content analysis results were evaluated using Minitab19 software with 95% significance level normality and two-way ANOVA / Tukey tests.

## Results

### Physical analysis in bee venom

The physical properties of the 25 bee venom samples were examined (Table 2). The bee venoms appeared in the form of a typical powder with a typical smell (resembling banana and pear). The colours of the bee venoms varied from light yellow to beige and light brown. According to the Turkish standard (TS) 13126, bee venom has a pungent smell, a bitter taste and a clear yellowish colour. It dries at room temperature, loses 30%–40% of its mass and crystallizes. The crystalline powder should have a pungent odour, a bitter taste, and an off-white colour. The physical properties of the bee venom samples were found to compatible with the standard.

**Table 2:** Physical properties of bee venoms.

Sample	Appearance	Color	Smell
Bee Venom 1	Typical Powder	Light Yellow	Typical
Bee Venom 2	Typical Powder	Beige	Typical
Bee Venom 3	Typical Powder	Light Brown	Typical
Bee Venom 4	Typical Powder	Light Yellow	Typical
Bee Venom 5	Typical Powder	Light Yellow	Typical
Bee Venom 6	Typical Powder	Light Yellow	Typical
Bee Venom 7	Typical Powder	Light Yellow	Typical
Bee Venom 8	Typical Powder	Light Yellow	Typical
Bee Venom 9	Typical Powder	Light Yellow	Typical
Bee Venom 10	Typical Powder	Light Yellow	Typical
Bee Venom 11	Typical Powder	Light Yellow	Typical
Bee Venom 12	Typical Powder	Light Yellow	Typical
Bee Venom 13	Typical Powder	Light Yellow	Typical
Bee Venom 14	Typical Powder	Light Brown	Typical
Bee Venom 15	Typical Powder	Light Yellow	Typical
Bee Venom 16	Typical Powder	Light Yellow	Typical
Bee Venom 17	Typical Powder	Light Yellow	Typical
Bee Venom 18	Typical Powder	Light Yellow	Typical
Bee Venom 19	Typical Powder	Light Brown	Typical
Bee Venom 20	Typical Powder	Light Yellow	Typical
Bee Venom 21	Typical Powder	Beige	Typical
Bee Venom 22	Typical Powder	Light Yellow	Typical
Bee Venom 23	Typical Powder	Light Yellow	Typical
Bee Venom 24	Typical Powder	Beige	Typical
Bee Venom 25	Typical Powder	Light Yellow	Typical

### Chemical analysis of bee venom

#### Moisture determination

The moisture values of the analysed bee venom samples ranged from 9.16 ± 0.10% to 10.56± 0.09% (Table 3). The results indicated no incompatibility in the physical properties of the venoms.

**Table 3:** Moisture content of the bee venoms (%).

Sample	Mean ± std
Bee Venom 1	9.85 ± 0.10 ^cdef^
Bee Venom 2	10.41 ± 0.18^ab^
Bee Venom 3	9.74 ± 0.18^efg^
Bee Venom 4	9.29 ± 0.10^hi^
Bee Venom 5	10.56 ± 0.09 ^a^
Bee Venom 6	9.16 ± 0.10^i^
Bee Venom 7	9.47 ± 0.14^ghi^
Bee Venom 8	10.2 ± 0.1^abc^
Bee Venom 9	9.82 ± 0.15^defg^
Bee Venom 10	9.86 ± 0.10^cdef^
Bee Venom 11	9.64 ± 0.07^efgh^
Bee Venom 12	10.2 ± 0.10^abc^
Bee Venom 13	9.74 ± 0.07^efg^
Bee Venom 14	9.59 ± 0.10^fgh^
Bee Venom 15	9.74 ± 0.10^efg^
Bee Venom 16	9.67 ± 0.19^efg^
Bee Venom 17	10.19 ± 0.06^bcd^
Bee Venom 18	9.77 ± 0.09^efg^
Bee Venom 19	9.57 ± 0.09^fgh^
Bee Venom 20	9.84 ± 0.08 ^cdefg^
Bee Venom 21	9.74 ± 0.08^efg^
Bee Venom 22	9.77 ± 0.08^efg^
Bee Venom 23	9.79 ± 0.19^efg^
Bee Venom 24	9.86 ± 0.09^cdef^
Bee Venom 25	10 ± 0.10^cde^

^1^The means indicated by different letters in the column exhibit statistically significant differences (p <0.05).

#### Determination of bee venom content using HPLC-UV

Using the HPLC-UV method, retention times were 23 minutes for melittin, 12 minutes for apamin and 18 minutes for phospholipase A2. The melittin content of the bee venom samples was in the range of 26.76%–51.85%. According to the TS 13126 Honeybee Venom standard, the melittin content should be between 40% and 50% in dry matter. The average amount of melittin in the present study was 40.57%, within the range of the standard.

The content of phospholipase A2 in bee venoms varied between 9.26% and 17.83%. According to the TS 13126 Honeybee Venom standard, phospholipase A2 content should be between 10%–12% in dry matter. The average content of phospholipase A2 in bee venoms in the present study was 13.67%.

The amount of apamin contained in the bee venoms ranged from 1.40% to 2.85%. According to TS 13126 Honeybee Venom standard, apamin content should be 1%–3% in dry matter. In this context, all venoms in this study complied with the standard.

The average apamin content of Anatolian bee venom samples in the present study was 2.12%. ANOVA / Tukey test results and grouping are given in Table 4. According to TS 13126 for Bee Venom, melittin should be between 40% and 50%. Analysis of the Anova/Tukey results for melittin revealed that venoms 6, 8, 10, 12, 17 and 21 were incompatible with the Turkish standard.

**Table 4:** Melittin, apamin, phospholipase A2 contents in bee venom.

Sample	Apamin (%)	Phospholipase (%)	Melittin (%)
Bee Venom 1	2.17 ± 0.1^x^	14.76 ± 0.1^x^	41.62 ± 0.1^x^
Bee Venom 2	2.30 ± 0.0^x^	13.86 ± 0.1^x^	46.16 ± 0.1^x^
Bee Venom 3	2.31 ± 0.0^x^	14.95 ± 0.1^x^	42.89 ± 0.1^x^
Bee Venom 4	2.83 ± 0.0^x^	15.35 ± 0.0^x^	43.69 ± 0.2^x^
Bee Venom 5	2.27 ± 0.0^x^	13.60 ± 0.1^x^	45.05 ± 0.5^x^
Bee Venom 6	1.69 ± 0.0^by^	9.96 ± 0.0^by^	37.37 ± 0.1^by^
Bee Venom 7	2.29 ± 0.0^ab^	14.75 ± 0.1^ab^	47.58 ± 0.3^ab^
Bee Venom 8	2.61 ± 0.1^a^	14.86 ± 0.1^a^	51.85 ± 0.1^a^
Bee Venom 9	2.10 ± 0.1^xd^	11.14 ± 0.0^xd^	40.02 ± 0.1^xd^
Bee Venom 10	1.99 ± 0.2^xd^	16.45 ± 0.1^xd^	36.71 ± 0.1^xd^
Bee Venom 11	2.29 ± 0.0^ab^	17.75 ± 0.1^ab^	47.48 ± 0.1^ab^
Bee Venom 12	1.73 ± 0.0^y^	10.41 ± 0.1^y^	31.12 ± 0.4^y^
Bee Venom 13	2.02 ± 0.1^xd^	10.86 ± 0.1^xd^	40.03 ± 0.3^xd^
Bee Venom 14	2.32 ± 0.2^x^	12.78 ± 0.1^x^	42.40 ± 0.1^x^
Bee Venom 15	2.85 ± 0.1^a^	20.95 ± 0.1^a^	45.04 ± 0.1^a^
Bee Venom 16	2.52 ± 0.0^x^	15.33 ± 0.3^x^	43.73 ± 0.1^x^
Bee Venom 17	2.13 ± 0.1^xd^	14.62 ± 0.1^xd^	35.65 ± 0.4^xd^
Bee Venom 18	2.42 ± 0.1^x^	12.70 ± 0.1^x^	46.33 ± 0.4^x^
Bee Venom 19	2.37 ± 0.0^ab^	14.30 ± 0.1^ab^	47.72 ± 0.2^ab^
Bee Venom 20	1.87 ± 0.0^x^	12.18 ± 0.2^x^	43.69 ± 0.2^x^
Bee Venom 21	1.40 ± 0.0^de^	9.27 ± 0.0^de^	26.76 ± 0.4^de^
Bee Venom 22	2.28 ± 0.0^x^	14.81 ± 0.0^x^	43.17 ± 0.2^x^
Bee Venom 23	1.98 ± 0.0^xd^	11.51 ± 0.0^xd^	42.06 ± 0.1^xd^
Bee Venom 24	2.17 ± 0.0^x^	14.76 ± 0.3^x^	41.62 ± 0.1^x^
Bee Venom 25	2.30 ± 0.0^x^	13.86 ± 0.0^x^	46.16 ± 0.1^x^
Average	2.12 ± 0.4	13.67 ± 2.6	40.57 ± 7.9

^1^ Means with different letters in the column for each feature differ statistically significantly (p <0.05). In the table, ’abc’ is expressed with the letter x and ’cde’ with the letter y.

Fructose, glucose and sucrose analyses were performed to measure the sugar content in the samples. The amount of glucose in the bee venoms ranged between 1% and 13.7%. Significant differences were observed in terms of glucose contents among the different samples.

Glucose, fructose and sucrose analysis results are given in Table 5. Bee venom samples were classified in terms of glucose, fructose and sucrose values. Bee Venom 5 exhibited the highest glucose, fructose and sucrose values. In order to evaluate the sugar analysis results in this study, total sugar contents are also given in Table 5.

**Table 5:** Glucose, fructose and sucrose contents in bee venom.

Sample	Glucose (%)	Fructose (%)	Sucrose (%)	Total sugar (%)
Bee Venom 1	1.50 ± 0.1^e^	1.23 ± 0.2^e^	0.03 ± 0.1^e^	2.73
Bee Venom 2	4.57 ± 0.1^bx^	5.93 ± 0.2^bx^	0.00 ± 0.0^bx^	10.50
Bee Venom 3	2.23 ± 0.0^x^	4.07 ± 0.2^x^	1.13 ± 0.2^x^	7.40
Bee Venom 4	2.50 ± 0.1^x^	3.30 ± 0.2^x^	1.17 ± 0.1^x^	7.00
Bee Venom 5	13.50 ± 0.2^a^	16.10 ± 0.2^a^	0.33 ± 0.2^a^	29.90
Bee Venom 6	7.57 ± 0.1^ab^	11.60 ± 0.1^ab^	0.27 ± 0.1^ab^	19.47
Bee Venom 7	6.00 ± 1.0^x^	2.10 ± 0.4^x^	0.07 ± 0.1^x^	8.10
Bee Venom 8	5.43 ± 2.2^bx^	5.83 ± 0.3^bx^	0.03 ± 0.1^bx^	11.27
Bee Venom 9	2.93 ± 2.0^x^	4.67 ± 0.3^x^	0.03 ± 0.1^x^	7.60
Bee Venom 10	3.37 ± 1.0^x^	3.47 ± 0.3^x^	0.10 ± 0.2^x^	6.83
Bee Venom 11	2.63 ± 1.6^bcd^	11.30 ± 0.6^bcd^	0.07 ± 0.1^bcd^	13.93
Bee Venom 12	5.33 ± 2.5^bc^	12.17 ± 0.9^bc^	0.00 ± 0.0^bc^	17.60
Bee Venom 13	2.67 ± 1.0^de^	2.10 ± 0.5^de^	0.00 ± 0.0^de^	4.97
Bee Venom 14	4.97 ± 2.9^bx^	4.50 ± 0.3^bx^	0.13 ± 0.1^bx^	9.67
Bee Venom 15	4.20 ± 1.2^bx^	5.93 ± 0.2^bx^	0.20 ± 0.1^bx^	10.23
Bee Venom 16	2.87 ± 1.7^x^	4.53 ± 0.9^x^	0.03 ± 0.1^x^	7.40
Bee Venom 17	6.27 ± 1.2^bx^	4.10 ± 0.1^bx^	0.20 ± 0.1^bx^	10.37
Bee Venom 18	5.70 ± 3.1^bx^	5.20 ± 0.1^bx^	0.00 ± 0.0^bx^	11.00
Bee Venom 19	4.77 ± 1.5^bx^	7.43 ± 0.2^bx^	0.23 ± 0.1^bx^	12.40
Bee Venom 20	3.33 ± 1.9^x^	4.77 ± 0.2^x^	0.03 ± 0.1^x^	8.10
Bee Venom 21	11.70 ± 0.6^bc^	5.50 ± 0.2^bc^	0.00 ± 0.0^bc^	17.30
Bee Venom 22	3.93 ± 2.9^x^	3.33 ± 0.1^x^	0.07 ± 0.1^x^	7.27
Bee Venom 23	4.37 ± 2.6^bx^	5.47 ± 0.4^bx^	0.03 ± 0.1^bx^	9.83
Bee Venom 24	4.20 ± 1.0^bx^	6.27 ± 0.8^bx^	0.03 ± 0.1^bx^	10.47
Bee Venom 25	3.23 ± 2.9^de^	2.27 ± 0.3^de^	0.07 ± 0.1^de^	5.50

^1^ Means with different letters in the column for each feature differ significantly (p <0.05). ’cde’ is expressed by the letter x in the table.

### Microbiological analysis

No microbial development was observed in 11 of the bee venom samples at total aerobic mesophilic bacteria counts. Microbial development observed between 1×10^2^ cfu / g-ml and 1×10^2^ cfu / g-ml and 1×10^2^ cfu / g-ml was observed in 14 venoms. In terms of *E*. *coli* counts, microbial development was observed at 1.2 ×10^2^ cfu / g-ml in bee venoms 1, 2, 3 and 5, while no microbial development observed in the other venom samples. In terms of *S*. *aureus* counts, no microbial development was detected in any of the 25 samples. The *C*. *albicans* count was 2×10^2^ cfu / g-ml in samples 1, 23 and 25. Total yeast count was higher in samples 2 and 5 (5 and 4 cfu/g-ml, respectively) (Table 6). Microbiological evaluation suggests that the microbial quality of bee venom may depend on several factors, such as the time and place of production.

**Table 6:** Microbiological analysis results for bee venoms.

Sample	Total Aerobic Mesophilic (cfu/g-ml)	*E*. *coli* (cfu/g-ml)	*S*. *aureus* (cfu/g-ml)	*C*. *albicans* (cfu/g-ml)	Total Mold (cfu/g-ml)	Total Yeast (cfu/g-ml)
Bee Venom 1	1×10^2^	2×10^2^	0	2×10^2^	0	0
Bee Venom 2	0	2×10^2^	0	0	2×10^2^	5×10^2^
Bee Venom 3	0	2×10^2^	0	0	1×10^2^	2×10^2^
Bee Venom 4	0	0	0	0	0	0
Bee Venom 5	5×10^2^	2×10^2^	0	1×10^2^	1×10^2^	4×10^2^
Bee Venom 6	0	0	0	0	0	3×10^2^
Bee Venom 7	1×10^2^	0	0	0	2×10^2^	0
Bee Venom 8	1×10^2^	0	0	1×10^2^	1×10^2^	0
Bee Venom 9	0	0	0	1×10^2^	1×10^2^	2×10^2^
Bee Venom 10	0	0	0	1×10^2^	0	2×10^2^
Bee Venom 11	1×10^2^	0	0	0	0	2×10^2^
Bee Venom 12	1×10^2^	0	0	0	2×10^2^	0
Bee Venom 13	0	0	0	1×10^2^	0	1×10^2^
Bee Venom 14	2×10^2^	0	0	0	1×10^2^	0
Bee Venom 15	2×10^2^	0	0	0	1×10^2^	2×10^2^
Bee Venom 16	2×10^2^	0	0	0	0	2×10^2^
Bee Venom 17	0	0	0	1×10^2^	1×10^2^	1×10^2^
Bee Venom 18	0	0	0	1×10^2^	0	0
Bee Venom 19	2×10^2^	0	0	0	0	2×10^2^
Bee Venom 20	1×10^2^	0	0	0	1×10^2^	0
Bee Venom 21	0	0	0	1×10^2^	1×10^2^	0
Bee Venom 22	0	0	0	0	0	2×10^2^
Bee Venom 23	1×10^2^	0	0	2×10^2^	1×10^2^	0
Bee Venom 24	1×10^2^	0	0	0	0	2×10^2^
Bee Venom 25	1×10^2^	0	0	2×10^2^	0	0

## Discussion

The average melittin content in this study was 40.57%. A previous study reported melittin content values of 29.6% for European bee venom (*A*. *mellifera mellifera* and *A*. *mellifera ligustica*) and 25.5% for Africanized bee venom (*A*. *mellifera scutellata*) samples [27]. The melittin content in this study is relatively higher than those values. This may be attributable to differences between the colonies studied [27]. African bees secrete lower amounts of melittin, due to their venom glands being smaller than those of European bees [27–30]. In another study, involving five bee venom samples collected from Romania a, melittin content ranged from 27.66% to 64.22% with an average of 58.45% [31]. The average melittin value in the present research was lower than in the Romanian study.

Another study investigated the Africanized honeybee venom profile and reported an average phospholipase A2 content of 12.2% [32]. The average phospholipase A2 content was rather higher in the present study of Anatolian bee venom. However, the study of five bee venom samples collected from Romania reported phospholipase A2 contents in the range of 10.96%–22.86%, with an average value of 15.13% [31]. This was lower than in the present study. The variation in venom composition may be due to various factors, including the method of venom collection and the species of honeybees involved. The bee venoms used in the Romanian study were collected from inside the beehive by stimulating the bees with electric current pulses. The venom collection frames were also located in the upper middle cavity of the upper body of the hive [31]. The objective was to obtain the highest level of efficiency in bee venom collection [31, 33]. In the present study, however, venom samples were collected by placing the platform at the entrance to the hive. Phospholipase A2 may also be affected by the conditions of collection since different methods result in different compositions of the final products [34]. In addition, Romanian honeybees (*A*. *carpatica*) and Turkish honeybees (*A*. *mellifera anatoliaca*) may have characteristic bee venom compositions, since the venom content depends on the species. *A*. *mellifera carpatica* is a valuable bee capable of producing honey and pollen of good quality [35]. The high phospholipase A2 content in Romanian samples may be attributable to Romania having a favourable geographical location, various relief forms, a continental climate, a wide variety of wild flora and cultivated agricultural products and experienced beekeepers [35].

The average apamin content of Anatolian bee venom samples in the present study was 2.12%. Melittin, phospholipase A2 and apamin contents of bee venom collected from Northeast Portugal were 86%, 13% and 2%, respectively, in another study [36]. The apamin and phospholipase A2 content was similar in Anatolian and Portuguese bee venom samples, although the melittin content differed. We attribute this to the difference in bee venom production seasons, since the total amount of protein varies with the age of the insect. A study analysing the total protein content of 7, 14, 21, 28, 35, and 40-day old *A*. *mellifera* L. worker bee venom glands in the summer and winter showed a higher level of venom extraction from older workers [37]. The study of five bee venom samples collected from Romania reported apamin content between 3.42% and 4.68%, with an average value of 4.09% [32].

The average apamin, phospholipase A2 and melittin contents in a study of 28 bee venom samples were 2.64%, 13.04% and 54.08%, respectively [38]. The phospholipase A2 content of Anatolian bee venom was higher than in that research, while the melittin and apamin contents were lower. Another study revealed apamin, phospholipase A2 and melittin contents in the ranges of 2%–3%, 10%–12% and 40%–50%, respectively [19]. The apamin and melittin contents of Anatolian bee venom in the present study were between these ranges, while the phospholipase A2 content was higher. The protein content of Anatolian bee venom appears to be consistent with the range of values in the previous literature.

The total sugar content of a quality bee venom should be less than 6.5% [1]. As shown in Table 5, the majority of the samples in this study had higher sugar contents then the recommended value. This may be due the bee venom sample collection method. On the other hand, it has been suggested that bee venom will not contain carbohydrates if collected in such a manner as to prevent contamination with pollen and nectar [39]. Bee venom can be collected by means of a platform placed either inside the hive, or at the entrance to the hive [40]. Since the bee venom samples in the present research were collected from the entrance to the hive, there is a strong possibility of pollen contamination. The high sugar found in the bee venom samples cannot therefore be regarded as in indicator of low quality since it results from the presence of pollen. Further studies are now required in order to validate the hypothesis, and new findings may improve bee venom collection methods.

The low microbial load in bee venom samples can be associated with the antimicrobial effect of the venom. In a study using the disk diffusion method to evaluate the antibacterial activity of bee venom against six gram-positive and gram-negative bacteria, namely *S*. *aureus*, *Salmonella typhimurium*, *E*. *coli* O157: H7, *Pseudomonas aeruginosa*, *Burkholderia mallei* and *Burkholderia pseudomallei*, antimicrobial activity was tested using three concentrations of bee venom and standard antibiotic (gentamicin) disks as positive controls. Bee venom was observed to exhibit antibacterial activity against *E*. *coli*, *S*. *aureus* and *S*. *typhimurium* at all three concentrations. The authors concluded that bee venom exerted higher antibacterial activity in the medium against *E*. *coli* than against the other two bacterial strains [41]. Given the environmental conditions and the nature of the bee venom, a high microbial load might be expected. However, the microbial load was almost non-existent for most samples. This shows that bee venom produces its own antimicrobial protection system.

Bee venom, like other natural toxins, is a chemical defence agent used by bees for self-protection. Phospholipase A2 has been shown to exhibit antibacterial activity and to represent a significant host defence molecule [42–45]. Phospholipase A2 destroys the living cell membrane through its powerful allergenic property [46]. In terms of the relationship between low microbial contamination and high phospholipase A2 content, it may be concluded that bee venom inhibits microbial growth through the presence of phospholipase A2.

## Conclusion

Bee venom samples were evaluated using physical, chemical and microbiological analyses in this study. The average melittin, apamin and phospholipase A2 contents were 40.57%, 2.12% and 13.67%, respectively. The results suggest that Anatolian bee venom has a higher phospholipase A2 content than that reported elsewhere in the literature. Apamin content was similar to that reported in other countries. The sugar content of the bee venom samples in this study were higher than those reported in the previous literature. We think that contamination of bee venom samples with pollen and nectar played a significant role in the high sugar content. Further studies investigating this association should now be performed in order to observe this relationship in greater detail. Melittin content was within the range of standard values. In terms of the *S*. *aureus* count, no microbial development was detected in any of the 25 bee venom samples. The low microbial load may be associated with the high phospholipase A2 level in the bee venom samples, due to its well-known antimicrobial properties.

## Supporting information

S1 File Statistical analysis of moisture content of the bee venoms. (DOCX)

S2 File Statistical analysis of melittin, apamin, phospholipase A2 contents in bee venom. (DOCX)

S3 File Statistical analysis of glucose, fructose and sucrose contents in bee venom. (DOCX)

## References

[1] Bogdanov S. (2016). Bee Venom: production, composition and quality. *The bee venom Book*.

[2] Özkök A. S. L. I. (2018). Türkiye’de Hızla Büyüyen Sektör: Arı Ürünlerine Genel Bir Bakış.

[3] Hider R. C. (1988). Honeybee venom: a rich source of pharmacologically active peptides. Endeavour, 12(2), 60–65. doi: 10.1016/0160-9327(88)90082-8 2458907

[4] Altıntaş L., & Bektaş N. (2019). Apiterapi: 1. Ari Zehri. *Uludag Bee Journal*, 19(1), 82–95.

[5] Mutlu C., Erbaş M., & Tontul S. A. (2017). Bal ve Diğer Arı Ürünlerinin Bazı Özellikleri ve İnsan Sağlığı Üzerine Etkileri. *Academic Food Journal/Akademik GIDA*, 15(1).

[6] Wehbe R., Frangieh J., Rima M., El Obeid D., Sabatier J. M., & Fajloun Z. (2019). Bee venom: Overview of main compounds and bioactivities for therapeutic interests. *Molecules*, 24(16), 2997. doi: 10.3390/molecules24162997 31430861 PMC6720840

[7] Abdu M. & Ali A. S. M. (2012). Studies on bee venom and its medical uses. *International Journal of Advancements in Research & Technology*, 1(2), 69–83.

[8] Hegazi A., Abdou A. M., El-Moez S., & Allah F. A. (2014). Evaluation of the antibacterial activity of bee venom from different sources. *World Applied Sciences Journal*, 30(3), 266–270.

[9] Hwang D. S., Kim S. K., & Bae H. (2015). Therapeutic effects of bee venom on immunological and neurological diseases. *Toxins*, 7(7), 2413–2421. doi: 10.3390/toxins7072413 26131770 PMC4516920

[10] Oršolić N., Šver L., Verstovšek S., Terzić S., & Bašić I. (2003). Inhibition of mammary carcinoma cell proliferation in vitro and tumor growth in vivo by bee venom. *Toxicon*, 41(7), 861–870. doi: 10.1016/s0041-0101(03)00045-x 12782086

[11] Yavari M., Salesi Z., Derakhti A., Azimzadeh S., Varpaei H. A., Esmaeili H., et al. (2020). Melittin and Breast Cancer: A Brief Review of the Evidence. *Journal of Nursing and Patient Safety*, 20(1), 1–7.

[12] Oršolić N. (2012). Bee venom in cancer therapy. *Cancer and Metastasis Reviews*, 31(1–2), 173–194. doi: 10.1007/s10555-011-9339-3 22109081

[13] Fletcher J. E., & Jiang M. S. (1993). Possible mechanisms of action of cobra snake venom cardiotoxins and bee venom melittin. *Toxicon*, 31(6), 669–695. doi: 10.1016/0041-0101(93)90375-s 8342168

[14] Wang C., Chen T., Zhang N., Yang M., Li B., Lü X., et al. (2009). Melittin, a major component of bee venom, sensitizes human hepatocellular carcinoma cells to tumor necrosis factor-related apoptosis-inducing ligand (TRAIL)-induced apoptosis by activating CaMKII-TAK1-JNK/p38 and inhibiting IκBα kinase-NFκB. *Journal of Biological Chemistry*, 284(6), 3804–3813. 10.1074/jbc.M807191200 19074436

[15] Hood J. L., Jallouk A. P., Campbell N., Ratner L., & Wickline S. A. (2013). Cytolytic nanoparticles attenuate HIV-1 infectivity. *Antivir Ther*, 18(1), 95–103. doi: 10.3851/IMP2346 22954649

[16] Jeong J. K., Moon M. H., Bae B. C., Lee Y. J., Seol J. W., & Park S. Y. (2011). Bee venom phospholipase A2 prevents prion peptide induced-cell death in neuronal cells. *International journal of molecular medicine*, 28(5), 867–873. doi: 10.3892/ijmm.2011.730 21701769

[17] Doery H. M. & Pearson J. E. (1964). Phospholipase B in snake venoms and bee venom. *Biochemical Journal*, 92 *(*3), 599–602. doi: 10.1042/bj0920599 5891197 PMC1206108

[18] Kim S. J., Park J. H., Kim K. H., Lee W. R., Pak S. C., Han S. M., et al. (2012). The protective effect of apamin on LPS/fat-induced atherosclerotic mice. *Evidence-Based Complementary and Alternative Medicine*, 2012. doi: 10.1155/2012/305454 22645626 PMC3357006

[19] Moreno M., & Giralt E. (2015). Three valuable peptides from bee and wasp venoms for therapeutic and biotechnological use: melittin, apamin and mastoparan. *Toxins*, 7(4), 1126–1150. doi: 10.3390/toxins7041126 25835385 PMC4417959

[20] Gu H., Han S. M., & Park K. K. (2020). Therapeutic Effects of Apamin as a Bee Venom Component for Non-Neoplastic Disease. *Toxins*, 12(3), 195. doi: 10.3390/toxins12030195 32204567 PMC7150898

[21] Lamy C., Goodchild S. J., Weatherall K. L., Jane D. E., Liégeois J. F., Seutin V., et al. (2010). Allosteric block of KCa2 channels by apamin. *Journal of Biological Chemistry*, 285(35), 27067–27077. doi: 10.1074/jbc.M110.110072 20562108 PMC2930706

[22] Feranchak A. P., Doctor R. B., Troetsch M., Brookman K., Johnson S. M., & Fitz J. G. (2004). Calcium-dependent regulation of secretion in biliary epithelial cells: the role of apamin-sensitive SK channels. *Gastroenterology*, 127(3), 903–913. doi: 10.1053/j.gastro.2004.06.047 15362045

[23] Lee W. R., Kim K. H., An H. J., Kim J. Y., Lee S. J., Han S. M., et al. (2014). Apamin inhibits hepatic fibrosis through suppression of transforming growth factor β1-induced hepatocyte epithelial–mesenchymal transition. *Biochemical and biophysical research communications*, 450(1), 195–201. doi: 10.1016/j.bbrc.2014.05.089 24878534

[24] Kim W. H., An H. J., Kim J. Y., Gwon M. G., Gu H., Lee S. J., et al. (2017). Apamin inhibits TNF-α-and IFN-γ-induced inflammatory cytokines and chemokines via suppressions of NF-κB signaling pathway and STAT in human keratinocytes. *Pharmacological Reports*, 69(5), 1030–1035. doi: 10.1016/j.pharep.2017.04.006 28958612

[25] Krell R. (1996). *Value-added products from beekeeping* (No. 124). Food & Agriculture Org.

[26] Samancı T., & Kekeçoğlu M. (2019). Comparison of Commercial and Anatolian Bee Venom in Terms of Chemical Composition. *Uludag Bee Journal*, 19(1).

[27] Schumacher M. J., Schmidt J. O., Egen N. B., & Dillon K. A. (1992). Biochemical variability of venoms from individual European and Africanized honeybees (Apis mellifera). *Journal of allergy and clinical immunology*, 90(1), 59–65. doi: 10.1016/s0091-6749(06)80011-4 1629508

[28] Pucca M. B., Cerni F. A., Oliveira I. S., Jenkins T. P., Argemí L., Sørensen C. V., et al. (2019). Bee updated: Current knowledge on bee venom and bee envenoming therapy. *Frontiers in immunology*, 10, 2090. doi: 10.3389/fimmu.2019.02090 31552038 PMC6743376

[29] Funari S. R. C., Zeidler P. R., Rocha H. C., & Sforcin J. M. (2001). Venom production by Africanized honeybees (Apis mellifera) and Africanized-European hybrids. *Journal of venomous Animals and Toxins*, 7(2), 190–198.

[30] Hoffman D. R., & Jacobson R. S. (1984). Allergens in hymenoptera venom XII: how much protein is in a sting. *Ann Allergy*, 52(4), 276–278. 6711914

[31] Ionete R. E., Dinca O. R., Tamaian R., & Geana E. I. (2013). Exploring Apis mellifera venom compounds using highly efficient methods. *Progress of Cryogenics and Isotopes Separation*, 16(2), 89–100.

[32] Junior R. S. F., Sciani J. M., Marques-Porto R., Junior A. L., Orsi R. D. O., Barraviera B., et al. (2010). Africanized honey bee (Apis mellifera) venom profiling: Seasonal variation of melittin and phospholipase A2 levels. *Toxicon*, 56(3), 355–362. doi: 10.1016/j.toxicon.2010.03.023 20403370

[33] Kokot Z. J., Matysiak J., Urbaniak B., & Dereziński P. (2011). New CZE-DAD method for honeybee venom analysis and standardization of the product. *Analytical and bioanalytical chemistry*, 399(7), 2487–2494. doi: 10.1007/s00216-010-4627-2 21221542 PMC3035776

[34] Bellik Y. (2015). Bee venom: its potential use in alternative medicine. *Anti-infective agents*, 13(1), 3–16.

[35] Popescu A., Dinu T. A., Stoian E., & Serban V. (2020). Bee honey production concentration in Romania in the eu-28 and global context in the period 2009–2018. *Scientific Papers*: *Management*, *Economic Engineering in Agriculture & Rural Development*, 20(3).

[36] Sobral F., Sampaio A., Falcão S., Queiroz M. J. R., Calhelha R. C., Vilas-Boas M., et al. (2016). Chemical characterization, antioxidant, anti-inflammatory and cytotoxic properties of bee venom collected in Northeast Portugal. *Food and Chemical Toxicology*, 94, 172–177. doi: 10.1016/j.fct.2016.06.008 27288930

[37] Abreu R. M. M., MORAES R., & Malaspina O. (2000). Histological aspects and protein content of Apis mellifera L. worker venom glands: the effect of electrical shocks in summer and winter. *Journal of Venomous Animals and Toxins*, 6(1), 87–98.

[38] Kokot Z. J., & Matysiak J. (2009). Simultaneous determination of major constituents of honeybee venom by LC-DAD. *Chromatographia*, 69(11–12), 1401–1405.

[39] Shkenderov S., & Produkti I. T. P. (1983). The Bee Products, in Bulgarian. *Zemizdat (Abstract in Honey bibliography)*, 1–238.

[40] Sanad R. E., & Mohanny K. M. (2013). The efficacy of a new modified apparatus for collecting bee venom in relation to some biological aspects of honey bee colonies. *J*. *Am*. *Sci*, 9(10), 177–182.

[41] Zolfagharian H., Mohajeri M., & Babaie M. (2016). Bee venom (Apis Mellifera) an effective potential alternative to gentamicin for specific bacteria strains: Bee venom an effective potential for bacteria. *Journal of pharmacopuncture*, 19(3), 225. doi: 10.3831/KPI.2016.19.023 27695631 PMC5043086

[42] Buckland A. G., & Wilton D. C. (2000). The antibacterial properties of secreted phospholipases A2. *Biochimica et Biophysica Acta (BBA)-Molecular and Cell Biology of Lipids*, 1488(1–2), 71–82. 11080678 10.1016/s1388-1981(00)00111-6

[43] Nevalainen T. J., Haapamäki M. M., & Grönroos J. M. (2000). Roles of secretory phospholipases A2 in inflammatory diseases and trauma. *Biochimica et Biophysica Acta (BBA)-Molecular and Cell Biology of Lipids*, 1488(1–2), 83–90. 11080679 10.1016/s1388-1981(00)00112-8

[44] Qu X. D., & Lehrer R. I. (1998). Secretory phospholipase A2 is the principal bactericide for staphylococci and other gram-positive bacteria in human tears. *Infection and immunity*, 66(6), 2791–2797. doi: 10.1128/IAI.66.6.2791-2797.1998 9596749 PMC108271

[45] Zhao H., & Kinnunen P. K. (2003). Modulation of the activity of secretory phospholipase A2 by antimicrobial peptides. *Antimicrobial Agents and Chemotherapy*, 47(3), 965–971. doi: 10.1128/AAC.47.3.965-971.2003 12604528 PMC149322

[46] Lad P. J., & Shier W. T. (1979). Activation of microsomal guanylate cyclase by a cytotoxic polypeptide: melittin. *Biochemical and Biophysical Research Communications*, 89(1), 315–321. doi: 10.1016/0006-291x(79)90980-x 38790

