# Correction: An evaluation of the chemical content and microbiological contamination of Anatolian bee venom

**DOI:** 10.1371/journal.pone.0269579

**Published:** 2022-06-02

**Authors:** The PLOS ONE Staff

The first author’s name is spelled incorrectly. The correct name is: Aslı Elif Tanuğur-Samancı. The correct citation is: Tanuğur-Samancı AE, Kekeçoğlu M (2021) An evaluation of the chemical content and microbiological contamination of Anatolian bee venom. PLOS ONE 16(7): e0255161. https://doi.org/10.1371/journal.pone.0255161https://doi.org/10.1371/journal.pone.0255161

The publisher apologizes for the error.
