# Supplementary material for: An evaluation of the chemical content and microbiological contamination of Anatolian bee venom
Source: PLoS One. 2021 Jul 22;16(7):e0255161. doi: 10.1371/journal.pone.0255161 (PMC8297878; doi:10.1371/journal.pone.0255161)
Supplement: S1 File — (DOCX) [file pone.0255161.s001.docx]

**————— 7.06.2021 08:41:29 ————————————————————**

**General Linear Model: Nem versus Analiz; Arı Zehri**

Factor Type Levels Values

Analiz fixed 3 1. Analiz; 2. Analiz; 3. Analiz

Arı Zehri fixed 25 BV1; BV10; BV11; BV12; BV13; BV14; BV15; BV16; BV17;

BV18; BV19; BV2; BV20; BV21; BV22; BV23; BV24; BV25;

BV3; BV4; BV5; BV6; BV7; BV8; BV9

Analysis of Variance for Nem, using Adjusted SS for Tests

Source DF Seq SS Adj SS Adj MS F P

Analiz 2 0,02314 0,02314 0,01157 0,84 0,438

Arı Zehri 24 7,17293 7,17293 0,29887 21,71 0,000

Error 48 0,66073 0,66073 0,01377

Total 74 7,85679

S = 0,117325 R-Sq = 91,59% R-Sq(adj) = 87,04%

Unusual Observations for Nem

Obs Nem Fit SE Fit Residual St Resid

4 10,2000 10,3988 0,0704 -0,1988 -2,12 R

7 9,5300 9,7288 0,0704 -0,1988 -2,12 R

46 9,8800 9,6621 0,0704 0,2179 2,32 R

67 9,9700 9,7755 0,0704 0,1945 2,07 R

R denotes an observation with a large standardized residual.

Grouping Information Using Tukey Method and 95,0% Confidence

Analiz N Mean Grouping

3. Analiz 25 9,844 A

1. Analiz 25 9,808 A

2. Analiz 25 9,805 A

Means that do not share a letter are significantly different.

Grouping Information Using Tukey Method and 95,0% Confidence

Arı

Zehri N Mean Grouping

BV5 3 10,560 A

BV2 3 10,410 A B

BV8 3 10,200 A B C

BV12 3 10,200 A B C

BV17 3 10,187 B C D

BV25 3 10,000 C D E

BV24 3 9,860 C D E F

BV10 3 9,860 C D E F

BV1 3 9,850 C D E F

BV20 3 9,843 C D E F G

BV9 3 9,817 D E F G

BV23 3 9,787 E F G

BV22 3 9,770 E F G

BV18 3 9,770 E F G

BV13 3 9,743 E F G

BV3 3 9,740 E F G

BV15 3 9,740 E F G

BV21 3 9,737 E F G

BV16 3 9,673 E F G

BV11 3 9,640 E F G H

BV14 3 9,590 F G H

BV19 3 9,567 F G H

BV7 3 9,473 G H I

BV4 3 9,290 H I

BV6 3 9,163 I

Means that do not share a letter are significantly different.
