# Supplementary material for: An evaluation of the chemical content and microbiological contamination of Anatolian bee venom
Source: PLoS One. 2021 Jul 22;16(7):e0255161. doi: 10.1371/journal.pone.0255161 (PMC8297878; doi:10.1371/journal.pone.0255161)
Supplement: S2 File — (DOCX) [file pone.0255161.s002.docx]

**————— 14.09.2020 14:08:55 ————————————————————**

Welcome to Minitab, press F1 for help.

**————— 15.09.2020 11:15:28 ————————————————————**

**General Linear Model: Veri versus Faktör; Analiz; Arı Zehri**

Factor Type Levels Values

Faktör fixed 3 Apamin; Fosfolipaz; Melitin

Analiz fixed 3 1. Analiz; 2. Analiz; 3. Analiz

Arı Zehri fixed 25 BV1; BV10; BV11; BV12; BV13; BV14; BV15; BV16; BV17;

BV18; BV19; BV2; BV20; BV21; BV22; BV23; BV24; BV25;

BV3; BV4; BV5; BV6; BV7; BV8; BV9

Analysis of Variance for Veri, using Adjusted SS for Tests

Source DF Seq SS Adj SS Adj MS F P

Faktör 2 58387,8 58387,8 29193,9 2059,96 0,000

Analiz 2 0,0 0,0 0,0 0,00 0,999

Arı Zehri 24 2251,1 2251,1 93,8 6,62 0,000

Error 196 2777,7 2777,7 14,2

Total 224 63416,7

S = 3,76458 R-Sq = 95,62% R-Sq(adj) = 94,99%

Unusual Observations for Veri

Obs Veri Fit SE Fit Residual St Resid

187 26,6300 34,2439 1,3515 -7,6139 -2,17 R

188 27,1900 34,2748 1,3515 -7,0848 -2,02 R

189 26,4600 34,2524 1,3515 -7,7924 -2,22 R

208 0,8800 -6,6552 1,3515 7,5352 2,14 R

209 0,8400 -6,6243 1,3515 7,4643 2,12 R

210 0,8500 -6,6467 1,3515 7,4967 2,13 R

211 13,9700 4,9019 1,3515 9,0681 2,58 R

212 14,2600 4,9328 1,3515 9,3272 2,65 R

213 14,5500 4,9104 1,3515 9,6396 2,74 R

214 15,1200 31,7972 1,3515 -16,6772 -4,75 R

215 14,8500 31,8281 1,3515 -16,9781 -4,83 R

216 14,9300 31,8057 1,3515 -16,8757 -4,80 R

R denotes an observation with a large standardized residual.

Grouping Information Using Tukey Method and 95,0% Confidence

Faktör N Mean Grouping

Melitin 75 40,571 A

Fosfolipaz 75 13,675 B

Apamin 75 2,118 C

Means that do not share a letter are significantly different.

Grouping Information Using Tukey Method and 95,0% Confidence

Analiz N Mean Grouping

2. Analiz 75 18,806 A

3. Analiz 75 18,783 A

1. Analiz 75 18,775 A

Means that do not share a letter are significantly different.

Grouping Information Using Tukey Method and 95,0% Confidence

Arı

Zehri N Mean Grouping

BV8 9 23,106 A

BV15 9 22,946 A

BV11 9 22,503 A B

BV7 9 21,539 A B

BV19 9 21,464 A B

BV2 9 20,772 A B C

BV4 9 20,624 A B C

BV16 9 20,530 A B C

BV18 9 20,483 A B C

BV5 9 20,308 A B C

BV22 9 20,088 A B C

BV3 9 20,053 A B C

BV1 9 19,517 A B C

BV20 9 19,248 A B C

BV14 9 19,168 A B C

BV23 9 18,518 A B C D

BV10 9 18,384 A B C D

BV9 9 17,756 A B C D

BV13 9 17,638 A B C D

BV17 9 17,468 A B C D

BV6 9 16,338 B C D E

BV12 9 14,421 C D E

BV25 9 14,326 C D E

BV21 9 12,474 D E

BV24 9 10,028 E

Means that do not share a letter are significantly different.

**————— 4.06.2021 10:36:07 ————————————————————**
