# Supplementary material for: An evaluation of the chemical content and microbiological contamination of Anatolian bee venom
Source: PLoS One. 2021 Jul 22;16(7):e0255161. doi: 10.1371/journal.pone.0255161 (PMC8297878; doi:10.1371/journal.pone.0255161)
Supplement: S3 File — (DOCX) [file pone.0255161.s003.docx]

**————— 14.09.2020 14:08:55 ————————————————————**

Welcome to Minitab, press F1 for help.

**————— 15.09.2020 11:15:28 ————————————————————**

**General Linear Model: Veri versus Faktör; Analiz; Arı Zehri**

Factor Type Levels Values

Faktör fixed 3 Fructose; Glucose; Sucrose

Analiz fixed 3 1. Analiz; 2. Analiz; 3. Analiz

Arı Zehri fixed 25 BV1; BV10; BV11; BV12; BV13; BV14; BV15; BV16; BV17;

BV18; BV19; BV2; BV20; BV21; BV22; BV23; BV24; BV25;

BV3; BV4; BV5; BV6; BV7; BV8; BV9

Analysis of Variance for Veri, using Adjusted SS for Tests

Source DF Seq SS Adj SS Adj MS F P

Faktör 2 1327,241 1327,241 663,620 150,17 0,000

Analiz 2 4,482 4,482 2,241 0,51 0,603

Arı Zehri 24 758,815 758,815 31,617 7,15 0,000

Error 196 866,128 866,128 4,419

Total 224 2956,666

S = 2,10215 R-Sq = 70,71% R-Sq(adj) = 66,52%

Unusual Observations for Veri

Obs Veri Fit SE Fit Residual St Resid

41 16,2000 12,0591 0,7547 4,1409 2,11 R

43 0,3000 6,7844 0,7547 -6,4844 -3,31 R

44 0,2000 6,5031 0,7547 -6,3031 -3,21 R

45 0,5000 6,4698 0,7547 -5,9698 -3,04 R

92 1,0000 5,8120 0,7547 -4,8120 -2,45 R

94 11,0000 7,0293 0,7547 3,9707 2,02 R

95 12,0000 6,7480 0,7547 5,2520 2,68 R

96 10,9000 6,7147 0,7547 4,1853 2,13 R

100 2,5000 7,2600 0,7547 -4,7600 -2,43 R

103 13,0000 8,1960 0,7547 4,8040 2,45 R

105 12,3000 7,8813 0,7547 4,4187 2,25 R

181 12,0000 7,1600 0,7547 4,8400 2,47 R

182 12,1000 6,8787 0,7547 5,2213 2,66 R

183 11,0000 6,8453 0,7547 4,1547 2,12 R

R denotes an observation with a large standardized residual.

Grouping Information Using Tukey Method and 95,0% Confidence

Faktör N Mean Grouping

Fructose 75 5,7267 A

Glucose 75 4,7907 B

Sucrose 75 0,1707 C

Means that do not share a letter are significantly different.

Grouping Information Using Tukey Method and 95,0% Confidence

Arı

Zehri N Mean Grouping

BV5 9 9,9778 A

BV6 9 6,4778 A B

BV12 9 5,8333 B C

BV21 9 5,7333 B C

BV11 9 4,6667 B C D

BV19 9 4,1444 B C D E

BV8 9 3,7667 B C D E

BV18 9 3,6333 B C D E

BV17 9 3,5222 B C D E

BV24 9 3,5000 B C D E

BV2 9 3,5000 B C D E

BV15 9 3,4444 B C D E

BV23 9 3,2889 B C D E

BV14 9 3,2000 B C D E

BV7 9 2,7222 C D E

BV20 9 2,7111 C D E

BV9 9 2,5444 C D E

BV3 9 2,4778 C D E

BV16 9 2,4778 C D E

BV22 9 2,4444 C D E

BV4 9 2,3222 C D E

BV10 9 2,3111 C D E

BV25 9 1,8556 D E

BV13 9 1,5889 D E

BV1 9 0,9222 E

Means that do not share a letter are significantly different.

**————— 4.06.2021 10:42:06 ————————————————————**
